# Model of theta frequency perturbations and contextual fear memory

**DOI:** 10.1002/hipo.23307

**Published:** 2021-02-03

**Authors:** Giuseppe Castegnetti, Daniel Bush, Dominik R. Bach

**Affiliations:** ^1^ Computational Psychiatry Research, Department of Psychiatry, Psychotherapy, and Psychosomatics University of Zurich Zurich Switzerland; ^2^ Institute of Cognitive Neuroscience University College London London UK; ^3^ Queen Square Institute of Neurology University College London London UK; ^4^ Wellcome Centre for Human Neuroimaging and Max Planck/UCL Centre for Computational Psychiatry and Ageing Research University College London London UK

**Keywords:** anxiety, anxiolytic, contextual fear, fear recall, theta rhythm

## Abstract

Theta oscillations in the hippocampal local field potential (LFP) appear during translational movement and arousal, modulate the activity of principal cells, and are associated with spatial cognition and episodic memory function. All known anxiolytics slightly but consistently reduce hippocampal theta frequency. However, whether this electrophysiological effect is mechanistically related to the decreased behavioral expression of anxiety is currently unclear. Here, we propose that a reduction in theta frequency affects synaptic plasticity and mnemonic function and that this can explain the reduction in anxiety behavior. We test this hypothesis in a biophysical model of contextual fear conditioning. First, we confirm that our model reproduces previous empirical results regarding the dependence of synaptic plasticity on presynaptic firing rate. Next, we investigate how theta frequency during contextual conditioning impacts learning. These simulations demonstrate that learned associations between threat and context are attenuated when learning takes place under reduced theta frequency. Additionally, our simulations demonstrate that learned associations result in increased theta activity in the amygdala, consistent with empirical data. In summary, we propose a mechanism that can account for the behavioral effect of anxiolytics by impairing the integration of threat attributes of an environment into the cognitive map due to reduced synaptic potentiation.

## 1 INTRODUCTION

Neural oscillations in the theta band are among the most prominent feature of the hippocampal local field potential (LFP; Buzsáki, 2002; O'Keefe & Nadel, 1978). Theta oscillatory activity is implicated in mnemonic function (Düzel, Penny, & Burgess, 2010), spatial cognition (Buzsáki & Moser, 2013), and behavior control under survival threat (Tovote, Fadok, & Lüthi, 2015). A large body of research has investigated antecedents of theta power or amplitude on the one hand and the mechanisms by which theta oscillations modulate the dynamics of individual neurons on the other. For example, hippocampal theta power increases in environments containing potential threats (Adhikari, Topiwala, & Gordon, 2010; Khemka, Barnes, Dolan, & Bach, 2017) and in response to threat‐predicting cues (Likhtik, Stujenske, A Topiwala, Harris, & Gordon, 2014; Seidenbecher, Laxmi, Stork, & Pape, 2003). The theta phase of individual spikes affects the magnitude and direction of ensuing synaptic plasticity (Hölscher, Anwyl, & Rowan, 1997; Hyman, Wyble, Goyal, Rossi, & Hasselmo, 2003), and hippocampal place cells exhibit a theta phase code for location (O'Keefe & Recce, 1993) suggestive of a role in spatial coding (Eichenbaum, Dudchenko, Wood, Shapiro, & Tanila, 1999).

The frequency of theta oscillations is ~4–12 Hz in rodent species (Colgin, 2013) and is known to increase with running speed during locomotion (Sławińska & Kasicki, 1998; Wells et al., 2013). Notably, theta frequency is slightly (0.5–2 Hz; McNaughton, Richardson, & Gore, 1986) but consistently reduced by anxiolytic drugs—compounds that alleviate rodent anxiety‐like behavior (for a review, see Nemeroff, 2003a), human anxiety‐like behavior (e.g., Bach, Korn, Vunder, & Bantel, 2018; Biedermann et al., 2017; Korn et al., 2016) and clinical anxiety in humans (McNaughton & Coop, 1991). In anesthetized rodents, the slowing of theta frequency is so robust that it has been proposed as a screening test for clinically effective anxiolytics (McNaughton, Kocsis, & Hajos, 2007; Yeung, Treit, & Dickson, 2012). Importantly, this frequency decrease is independent of the relationship between theta frequency and running speed (Monaghan, Chapman, & Hasselmo, 2017; Wells et al., 2013). Theta slowing has been observed for all known anxiolytic agents (McNaughton & Coop, 1991). This includes barbiturates and benzodiazepines (McNaughton et al., 1986), sometimes categorized together as “classical anxiolytics” (Riva, Gaudio, & Dakanalis, 2015), as well as many “novel” anxiolytic agents of more recent discovery such as buspirone (Coop & McNaughton, 1991), the selective serotonin reuptake inhibitor fluoxetine (Munn & McNaughton, 2008), the tricyclic antidepressant imipramine (Zhu & McNaughton, 1995), and the GABA agonist pregabalin (Siok, Taylor, & Hajós, 2009). Theta slowing is also seen in drugs that have anxiolytic properties but are not primarily used to that end, such as somatostatin (Engin, Stellbrink, Treit, & Dickson, 2008).

Strikingly, despite their common effects on theta frequency and behavior, the molecular targets of these various anxiolytics are clearly distinct. Specifically, barbiturates and benzodiazepines interact with the neurotransmission of γ‐aminobutyric acid (GABA) and have been shown to affect only specific GABA‐A receptor subunits (Macdonald & Olsen, 1994); and pregabalin increases GABA levels (Nemeroff, 2003b). In contrast, buspirone, selective serotonin reuptake inhibitors, and tricyclic antidepressants target the serotonergic (5‐hydroxytryptamine; 5‐HT) system, albeit via different pharmacological mechanisms (Hiemke & Hartter, 2000; Mahmood & Sahajwalla, 1999). This suggests that the behavioral expression of anxiolysis is more closely related to a decrease in theta frequency than to the proximal molecular mechanism of action. Nonetheless, these observations are merely correlational, and there is no data to confirm or refute a suggestion that anxiolytic theta slowing is mechanistically related to a reduction of anxiety‐like behavior.

In this paper, we sought to provide a possible mechanistic link between changes in theta frequency and anxiety‐like behavior using a biophysical proof‐of‐concept model. From the many assays of anxiety‐like behavior, we chose to simulate contextual fear conditioning (Grillon & Ernst, 2020; Likhtik et al., 2014; Maren & Hobin, 2007; Phillips & LeDoux, 1992), which is well amenable to computational modeling due to its high level of experimental control. Contextually conditioned freezing is consistently reduced by anxiolytic drugs (Ehrlich et al., 2009; Luyten, Vansteenwegen, Van Kuyck, Gabriëls, & Nuttin, 2011; Sanger & Joly, 1985). Our model system is composed of a population of putative amygdalar neurons that respond selectively to noxious stimuli and receive feed‐forward excitation from a population of theta‐modulated hippocampal place cells (Jung, Wiener, & McNaughton, 1994; O'Keefe & Nadel, 1978; Ranck, 1973). Building on a standard model of synaptic plasticity, we show that a small reduction in theta frequency substantially reduces the potentiation of these feedforward connections during simulated contextual fear conditioning, in line with recent empirical work that demonstrated a correlation between theta frequency and spatial learning (Young, Ruan, & McNaughton, 2020). This leads to reduced expression of contextual fear (i.e., reduced firing rates in simulated amygdalar neurons) during subsequent exposure to the conditioned context. To validate our model, we finally show that it can also account for the occurrence of amygdalar theta oscillations upon presentation of a conditioned stimulus, as has been observed experimentally (Lesting et al., 2011; Likhtik et al., 2014; Seidenbecher et al., 2003). In summary, we demonstrate a network‐level mechanism that is capable of accounting for the common impact of anxiolytic drugs on anxiety‐like behavior via their impact on theta frequency, despite their heterogeneous molecular targets.

## 2 METHODS

### 2.1 Dendritic spine model

To investigate the effect of theta frequency on synaptic plasticity and contextual fear conditioning, we simulate postsynaptic dendritic spines on a population of *N* “fear cells” that receive synaptic input from a population of *M* hippocampal place cells. These fear cells are active whenever the simulated agent perceives a noxious stimulus, analogous to neurons in the lateral nucleus of the amygdala (LA; Paré & Collins, 2000; Romanski, Clugnet, Bordi, & LeDoux, 1993), while place cells produce theta modulated spike trains whenever the animal is located at a particular location within the environment (O'Keefe & Nadel, 1978; Thompson & Best, 1989). In these simulations, we assume that during salient or novel experiences, acetylcholine (ACh) is released into the hippocampus to promote learning by enhancing synaptic plasticity but reducing the strength of intrahippocampal connections that might generate recall. Conversely, during familiar experiences, levels of ACh in the hippocampus are low, promoting recall by enhancing intrahippocampal connections but reducing synaptic plasticity that might disrupt existing associations. This relationship is supported by empirical data (Douchamps, Jeewajee, Blundell, Burgess, & Lever, 2013; Hasselmo, 2006). For example, intracerebral administration of cholinergic antagonists into either the hippocampus or basolateral amygdala (BLA) suppresses the acquisition of conditioned fear (Wilson & Fadel, 2017), as does the optogenetic inhibition of cholinergic activity in the BLA during training (Jiang et al., 2016). Conversely, optogenetic enhancement of cholinergic neurons in the medial septum—the main ACh input to the hippocampus—enhances contextual fear conditioning (Hersman et al., 2017).

The membrane potential at each dendritic spine is a linear sum of two components: excitatory postsynaptic potentials (EPSPs) generated by input from hippocampal place cells, and backpropagating action potentials (BPAPs) from the soma. Each of these inputs generates depolarization away from the resting membrane potential *V*_*r*_:

(1) $$V_{s}\left(t\right)=V_{r}+\text{EPSP}\left(t\right)+\text{BPAP}\left(t\right)$$

EPSPs generated by an input spike at time *t*_*i*_ are modeled as the sum of two exponential functions with time constants $\tau_{1}^{\mathrm{ep}}$ and $\tau_{2}^{\mathrm{ep}}$, respectively, modulated by the presence of acetylcholine ([ACh]; Hasselmo, 2006), and a normalization parameter *s* chosen to produce peak depolarization of 8 mV:

(2) $$\text{EPSP}\left(t\right)=\left(1-\left[\mathrm{ACh}\right]\right)\cdot s\cdot \sum_{i}\left(e^{-\frac{t-t_{i}}{\tau_{1}^{\mathrm{ep}}}}-e^{-\frac{t-t_{i}}{\tau_{2}^{\mathrm{ep}}}}\right)$$

The choice of the parameter *s* is in line with empirical reports (Rosenkranz, 2012), although we note that is makes little qualitative difference to the results.

BPAPs generated by an output spike at time *t*_0_ are modeled as the sum of two exponential functions with time constants $\tau_{f}^{\mathrm{bs}}$ and $\tau_{s}^{\mathrm{bs}}$ that correspond to a fast spike and slower after‐depolarising potential, respectively (following Shouval, Bear, & Cooper, 2002). The relative amplitude of the fast and slow BPAP components is dictated by the parameters $I_{f}^{\mathrm{bs}}$ and $I_{s}^{\mathrm{bs}}$:

(3) $$\text{BPAP}\left(t\right)=100\cdot \left(I_{f}^{\mathrm{bs}}e^{-\frac{t-t_{0}}{\tau_{f}^{\mathrm{bs}}}}+I_{s}^{\mathrm{bs}}e^{-\frac{t-t_{0}}{\tau_{s}^{\mathrm{bs}}}}\right)$$

The value of the parameters of the dendritic spine model used in the simulations are summarized in Table 1.

**TABLE 1:** Model parameters used to simulate the contextual conditioning protocols (Shouval et al., 2002)

Parameter	Description	Value
*τ*	Time constant of the neuronal membrane	20 ms
*v*_*rest*_	Neuronal resting potential	−65 mV
v^*^	Neuronal firing threshold	−55 mV
*v*_*reset*_	Neuronal reset potential	−75 mV
*λ*	Synaptic strength decay constant	0.1
$\tau_{1}^{\mathrm{ep}}$	Slow EPSP time constant	50 ms
$\tau_{2}^{\mathrm{ep}}$	Fast EPSP time constant	5 ms
*α*_1_	Parameter used in the definition of Ω (Equation ((5))	0.35
*α*_2_	Parameter used in the definition of Ω (Equation ((5))	0.55
*β*_1_	Parameter used in the definition of Ω (Equation ((5))	80
*β*_2_	Parameter used in the definition of Ω (Equation ((5))	80
*P*_1_	Parameter used in the definition of *η* (Equation (7))	0.1 s
*P*_2_	Parameter used in the definition of *η* (Equation (7))	*P*_1_ /10^−4^
*P*_3_	Parameter used in the definition of *η* (Equation (7))	3
*P*_4_	Parameter used in the definition of *η* (Equation (7))	1 s
*τ*_Ca_	Calcium time constant	50 ms
*P*_0_	Probability of NMDAr opening after action potential	0.5
*G*_*NMDA*_	NMDAr conductance	−1/500 [μM/(ms · mV)]
*I*_*f*_	Fast NMDAr current component intensity	0.5
*I*_*s*_	Slow NMDAr current component intensity	0.5
*τ*_*f*_	Fast NMDAr current component time constant	50 ms
*τ*_*s*_	Slow NMDAr current component time constant (NMDAr)	200 ms
*V*_*r*_	Reversal potential for calcium	130 mV
*A*_*θ*_	Amplitude of the intracellular theta oscillation	3 mV
$I_{f}^{\mathrm{bs}}$	Fast BPAP component intensity	0.75
$I_{s}^{\mathrm{bs}}$	Slow BPAP component intensity	0.25
$\tau_{f}^{\mathrm{bs}}$	Fast BPAP component time constant	3 ms
$\tau_{s}^{\mathrm{bs}}$	Slow BPAP component time constant	25 ms
*K*	Number of simulated input spikes	900
*r*_*safe*_	Fear cells' firing rate in the safe compartment	0.85 Hz
*r*_*threat*_	Fear cells' firing rate in the threatening compartment	1.85 Hz

### 2.2 Synaptic plasticity model

Consistent with previous empirical (Bear, Cooper, & Ebner, 1987; Lisman, 1989) and theoretical studies, we assume that activity‐dependent changes in synaptic strength *W* at each dendritic spine are governed by intracellular Calcium concentration [*Ca*^2+^], in accordance with the calcium control hypothesis (Shouval et al., 2002). The values of the parameters of the synaptic plasticity model described below are listed in Table 1.

Calcium concentration in the dendritic spine increases in proportion to the current influx through NMDA receptors, *I*_*NMDA*_, which does not contribute to the membrane potential, and subsequently decays with a constant *τ*_*Ca*_:

(4) $$\frac{d\left[Ca^{2+}\right]}{\mathrm{dt}}=I_{\text{NMDA}}\left(t\right)-\frac{1}{\tau_{\mathrm{Ca}}}\left[Ca^{2+}\right]$$

NMDA currents, in turn, are governed by the channel opening probability *P*_0_, the maximum channel conductance *G*_*NMDA*_, the time constants of rising and decay *τ*_*f*_ and *τ*_*s*_, respectively, and a voltage‐dependent term characterizing the blockade of NMDA channels by magnesium *H*(*V*_*s*_), where [*Mg*^2+^] represents extracellular magnesium concentration and $E_{{\mathrm{Ca}}^{2+}}$ the calcium reversal potential:

(5) $$I_{\text{NMDA}}\left(t\right)=P_{0}\cdot G_{\text{NMDA}}\cdot \left(I_{f}e^{-\frac{t-t_{i}}{\tau_{f}}}+I_{s}e^{-\frac{t-t_{i}}{\tau_{s}}}\right)\cdot H\left(V_{s}\right)$$

(6) $$H\left(V_{s}\right)=\frac{V_{s}-E_{{\mathrm{Ca}}^{2+}}}{1+e^{-0.062V_{s}}\left[Mg^{2+}\right]/3.57}$$

Finally, the magnitude and direction of changes in synaptic strength are governed by a non‐linear function of calcium concentration Ω([*Ca*^2+^]), which is modulated by a learning rate *η*([*Ca*^2+^]) and the presence of ACh (Hasselmo, 2006):

(7) $$\frac{\mathrm{dW}}{\mathrm{dt}}=\eta \left(\left[Ca^{2+}\right]\right)\cdot \Omega \left(\left[Ca^{2+}\right]\right)\cdot \left[\mathrm{ACh}\right]-\lambda$$

(8) $$\Omega \left(\left[Ca^{2+}\right]\right)=0.25+s\left(\left[Ca^{2+}\right]-\alpha_{2},\beta_{2}\right)-0.25s\left(\left[Ca^{2+}\right]-\alpha_{1},\beta_{1}\right)$$

(9) $$s\left(x,\beta \right)=\frac{e^{\mathrm{\beta x}}}{e^{\mathrm{\beta x}}+1}$$

(10) $$\eta \left(\left[Ca^{2+}\right]\right)={\left(\frac{P_{1}}{P_{2}+{\left(\left[Ca^{2+}\right]\right)}^{P_{3}}}+P_{4}\right)}^{-1}$$

### 2.3 Stimulation protocols

First, to confirm that the plasticity model above can replicate changes in synaptic weight observed in vitro (e.g., O'Connor, Wittenberg, & Wang, 2005), we subjected dendritic spines to “tetanic stimulation” with trains of 100 spikes delivered at varying frequency *f*_*θ*_ in the absence of any output spiking activity, and with *λ* = 1 and *s* = 1.45 (Shouval et al., 2002).

**FIGURE 1:**
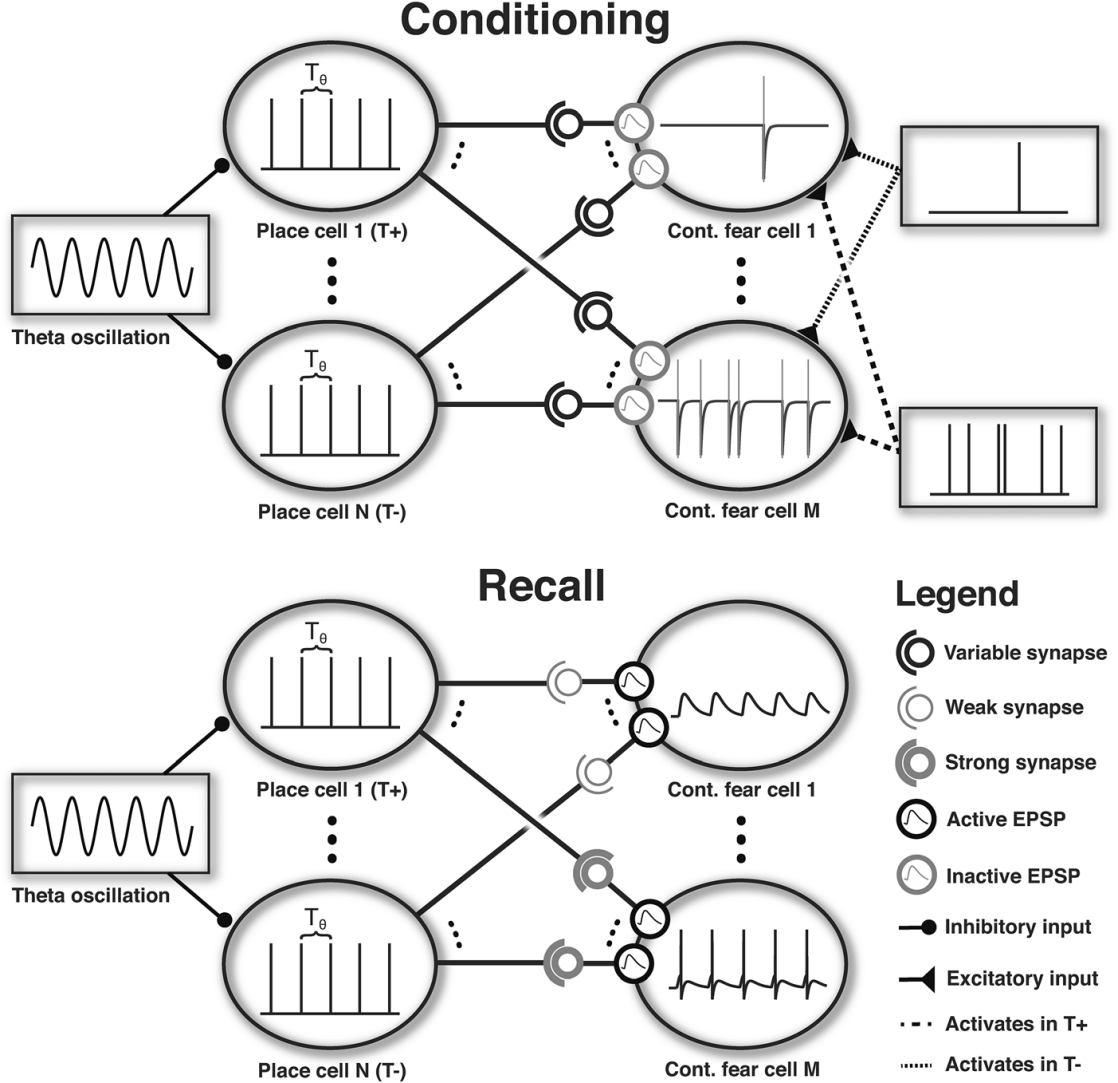
Schematic representation of the network configuration during conditioning and recall. Top—During conditioning, high levels of ACh inhibit EPSPs but support plasticity. Each fear cell receives location‐dependent inputs from the hippocampus, which activate NMDA receptors, and produce Poisson spike trains that reflect the absence or presence of noxious stimuli in the safe and threatening compartments, respectively. Bottom—During recall, low levels of ACh enhance EPSPs while inhibiting further synaptic plasticity. Noxious stimuli are no longer delivered, and the activity of fear cells is thus determined by place cell inputs and the synaptic weights induced by prior conditioning. As a result, place cells active in T^+^ elicit stronger activity in fear cells. In both panels, T_θ_ indicate the period of the theta rhythm

Next, we sought to examine the impact of changes in theta frequency on learning during a simulated contextual fear conditioning paradigm. We divided the *M* hippocampal place cells into two subpopulations that were each active in one of two contexts: a threatening compartment *T*^+^ and a safe compartment *T*^−^ (see Figure 1). While active, place cells fired rhythmic, inhomogeneous Poisson spike trains according to the rate function *r*_*PC*_, with theta frequency *f*_*θ*_ varying across simulations and a gain factor *K* set to produce an average of one spike per oscillatory cycle:

(11) $$r_{\mathrm{PC}}=K\cdot \frac{1+\sin\left(2\pi f_{\theta }t\right)}{\sum_{0}^{t_{\max}}1+\sin\left(2\pi f_{\theta }t\right)}$$

While in the safe compartment *T*^−^, fear cells fired homogenous Poisson spike trains with a rate of *r*_*safe*_; while in the threatening compartment *T*^+^, fear cells fired homogenous Poisson spike trains with an increased rate of *r*_*threat*_. During conditioning, the simulated animal spent a time interval corresponding to 100 theta periods in each compartment (Likhtik et al., 2014; Maren & Hobin, 2007; Phillips & LeDoux, 1992), and the level of acetylcholine was set to [ACh] = 1 to promote synaptic plasticity but eliminate the recall of previously encoded associations by reducing EPSP amplitude (Equations (2) and (7)).

After conditioning, we tested the simulated agent's ability to recall contextual fear. The level of acetylcholine was set to [ACh] = 0 to eliminate further synaptic plasticity but promote the recall of previously encoded associations by enhancing EPSP amplitude. Hippocampal theta frequency was set to *f*_*θ*_ = 5 Hz and the simulated agent spent another interval of 25 theta periods in each compartment. In this case, we were interested in the output firing rate of fear cells generated by input from the corresponding place cell population, assuming that elevated fear cell firing rate elevates levels of freezing behavior, a typical behavioral measure of fear. We assumed that the somatic membrane potential of fear cells *V*_*soma*_ was equal to the average membrane potential of all dendritic spines, and that output spikes were fired whenever the somatic membrane potential exceeded a threshold *V*_*thr*_, after which the membrane potential of all spines was set to the reset membrane potential *V*_*reset*_. We quantified the probability of freezing in each compartment (*T*^+^ or *T*^−^) as the fraction of 100 simulations in which the average firing rate of the fear cell population exceeded the threshold *r*_*freeze*_ =1.5 Hz.

### 2.4 Postsynaptic spike train analysis

Finally, we sought to quantify the theta modulation of output fear cell spike trains before and after the simulated fear conditioning protocol described above. To do so, we first computed the temporal auto‐correlation of spikes fired by each fear cell in 10 ms bins for lags of up to 1 s. We subsequently computed the fast Fourier transform of the mean‐normalized temporal auto‐correlation for frequencies up to 50 Hz and smoothed the resulting power spectra with a Gaussian kernel of 2 Hz width.

## 3 RESULTS

### 3.1 Synaptic plasticity model

**FIGURE 2:**
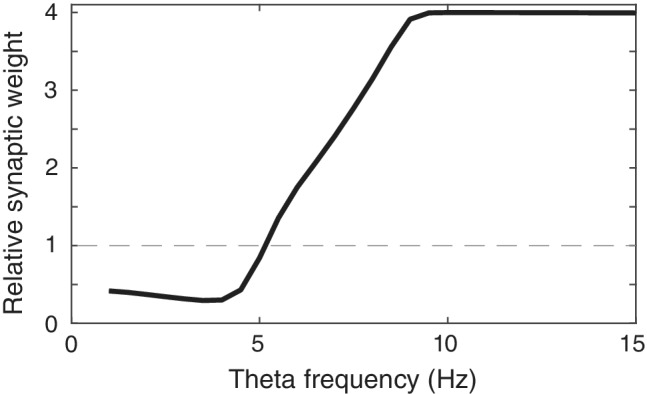
Dependence of synaptic strength on theta frequency during simulation of a presynaptic rate‐induced plasticity protocol

Despite their different molecular mechanisms of action, anxiolytic drugs generate both a reduction in anxiety behavior, including contextual fear conditioning (Ehrlich et al., 2009; Luyten et al., 2011; Sanger & Joly, 1985), as well as a small but consistent decrease in hippocampal theta frequency. Here, we sought to examine whether the latter phenomenon could potentially explain the altered behavioral phenotype. To this end, we built a neural model of theta modulated hippocampal place cells projecting to amygdalar “fear cells” through synapses that followed a standard calcium‐dependent plasticity rule (Shouval et al., 2002). First, to establish that this plasticity rule could account for the empirically observed dependence of synaptic modifications on the frequency of trains of afferent stimuli (Dudek & Bear, 1992, 1993; Mulkey & Malenka, 1992), we simulated a presynaptic “tetanic stimulation” protocol at different stimulation frequencies (see Methods). Consistent with previous empirical (O'Connor et al., 2005) and theoretical (Shouval et al., 2002) data, synaptic strength increased as a function of presynaptic stimulation frequency (Figure 2). Interestingly, the greatest change in synaptic strength was observed approximately between 4 and 9 Hz, that is, within a frequency band that roughly overlaps with rodent hippocampal theta frequency (Colgin, 2016).

### 3.2 Contextual fear conditioning

**FIGURE 3:**
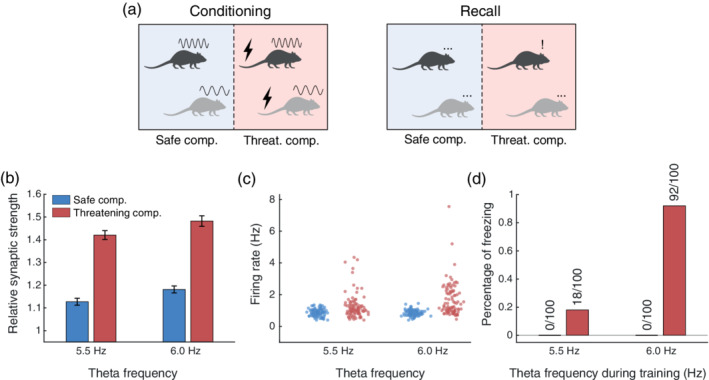
(a) Schematic of the simulated experimental protocol. Conditioning: High and low frequency sinusoids above the rodent's head represent conditioning at either high or low theta frequency, respectively, whereas the bolt symbol indicates the presence of noxious stimuli. Recall: The symbols above the rodent's head illustrate the behavior predicted by the model, with the three dots indicating no behavioral response and the exclamation mark a freezing response to the contextual cue. (b) Relative average synaptic strength obtained after simulating contextual conditioning during epochs of theta activity at 5.5 or 6 Hz in the threatening and safe compartment; error bars represent *SEM*. (c) Firing rate distribution of fear cells during recall at both theta frequencies in both compartments. (d) Percentage probability of freezing over 100 simulations

Next, to quantify whether this dependency was sufficient to explain the behavioral effect of anxiolytics, we simulated a standard contextual fear conditioning protocol (Likhtik et al., 2014; Figure 1, Top). During conditioning, our simulated agent explored an arena divided into two parts: a safe compartment and a threatening compartment (Figure 3a). Different subpopulations of theta‐modulated hippocampal place cells were active in each compartment, while fear cells fired Poisson spike trains at a higher rate in the threatening compartment than in the safe compartment (Paré & Collins, 2000; Romanski et al., 1993). During acquisition, high levels of ACh supported synaptic plasticity and inhibited the recall of existing associations (Hasselmo, 2006). Hippocampal theta frequency was either set to a baseline value of 6 Hz, or a reduced value of 5.5 Hz to reflect the small but significant reduction in frequency associated with the administration of anxiolytics. While conditioning increased the strength of synaptic inputs from place cells active in both compartments and at both theta frequencies, potentiation was stronger after conditioning at 6 Hz (consistent with the tetanic stimulation results illustrated in Figure 2) and when post‐synaptic firing rates were greater in the threatening compartment (Figure 3b).

### 3.3 Contextual fear recall

Following conditioning, we assessed the behavioral expression of learned anxiety—here modeled as contextual fear—during a subsequent recall phase in which noxious stimuli were no longer present (Figure 1, Bottom). In this case, the simulated agent was again exposed to each compartment of the conditioning arena, with the level of ACh reduced to promote recall and inhibit further synaptic plasticity. In this case, activity in the fear cell population was generated by input from theta modulated hippocampal place cells, and output firing rates reflected the relative strength of synaptic inputs generated during the acquisition phase. Hence, the firing rate of fear cells was greater in the threatening compartment, and greater following conditioning with a higher theta frequency (i.e., in the absence of anxiolytics, Figure 3c).

To relate this result to behavior, we sought to quantify the probability of freezing in each condition, which we assumed to occur whenever the average firing rate of the fear cell population exceeded a threshold. Under this assumption, the simulated agent exhibited no freezing in the safe compartment, regardless of theta frequency during conditioning. Conversely, in the threatening compartment, freezing occurred in 92% of simulations after conditioning with a theta frequency of 6 Hz, but only in 18% of the simulations after conditioning at 5.5 Hz (Figure 3d). We emphasize that any choice of freezing threshold and theta frequencies (within the 4–9 Hz range), would produce qualitatively similar results: a small reduction in theta frequency (as small as 0.5 Hz) is sufficient to significantly reduce synaptic potentiation, and therefore impair the acquisition of conditioned contextual fear when the freezing threshold is set accordingly. Below this range, no synaptic potentiation occurs; above this range, synaptic potentiation is saturated. In either case, changes in frequency have no effect. In summary, our model predicts a substantial difference between the behavioral expression of anxiety following contextual fear conditioning with hippocampal theta frequencies that varied by only 0.5 Hz. This suggests that a decrease in theta frequency within the range generated by the administration of anxiolytics can account for a significant reduction in anxiety in a model of contextual fear conditioning.

### 3.4 Model validation: Postsynaptic theta

**FIGURE 4:**
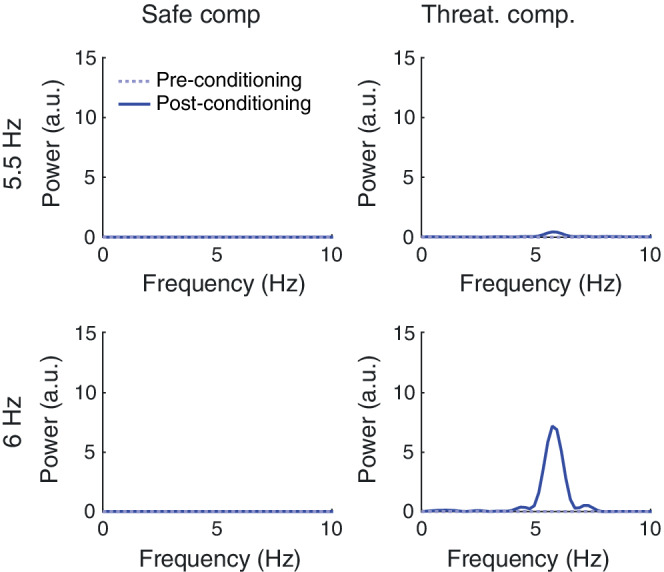
Power spectra of fear cell output spike trains in the safe (left) or threatening compartment (right). The solid cyan line and the blue dashed line indicate the spectrum before and after conditioning, respectively, with either low (5.5 Hz; top) or high (6.0 Hz; bottom) hippocampal theta frequency [Color figure can be viewed at wileyonlinelibrary.com]

Previous empirical studies have revealed theta‐band oscillatory activity in the rodent lateral amygdala (LA) during the presentation of conditioned threat cues, and these oscillations are in phase coherence with ongoing hippocampal theta oscillations (Likhtik et al., 2014; Seidenbecher et al., 2003). To validate our model, we sought to demonstrate that the simulated fear cells (which are analogous to LA neurons; Romanski et al., 1993) also exhibit this property, as their output spike trains are primarily dictated by increased synaptic input from theta modulated hippocampal place cells after the acquisition of contextual fear. We computed power spectra for fear cell spike trains in each condition (see Methods) and found a peak in the theta frequency band in the threatening compartment following conditioning at 6.0 Hz. At 5.5 Hz, we found a similar peak but with amplitude lower by an order of magnitude (Figure 4). Hence, our model can account for the appearance of theta‐band oscillations in LA following contextual fear conditioning, consistent with empirical observations (Likhtik et al., 2014; Seidenbecher et al., 2003).

## 4 DISCUSSION

In spite of their different molecular mechanisms of action, all known anxiolytic drugs reduce the frequency of hippocampal theta oscillations. This reduction is usually small—between 0.5 and 2 Hz (McNaughton et al., 1986)—but its specificity to anxiolytics led researchers to regard it as a possible test for anxiolytic agents (McNaughton et al., 2007; Yeung et al., 2012). In this study, we investigated whether this theta frequency reduction could mechanistically explain the behavioral effect of these drugs on anxiety behavior. We based our analysis on contextual fear conditioning, a paradigm in which conditioned freezing is reduced by anxiolytic drugs (Ehrlich et al., 2009; Luyten et al., 2011; Sanger & Joly, 1985). Our simulations demonstrate that even small frequency reductions can have a significant effect on synaptic plasticity, sufficient to disrupt the contextual association between environment and threat.

Put differently, our model predicts that increasing theta frequency during contextual threat learning would increase the strength of these associations, thereby increasing the behavioral expression of contextual fear. Two factors contribute to this effect. First, the relationship between synaptic potentiation and the frequency of presynaptic activity is steep in a frequency band that approximately corresponds to rodent theta (4–9 Hz; Figure 2 in the main text; see also Figure 3b in Shouval et al., 2002). Therefore, small increases in hippocampal theta frequency can have a substantial effect on the synaptic potentiation of (presynaptic) hippocampal place cell inputs to (postsynaptic) neurons in the amygdala (“fear cells”). Second, freezing behavior (the measure of conditioned fear) is assumed to be elicited when average firing rates in the amygdala exceed some threshold, which is less likely if synaptic inputs from hippocampal place cells encoding context are weaker. Hence, small reductions in theta frequency can substantially reduce the strength of inputs to the amygdala, decreasing firing rates in the amygdala and thus the probability of freezing (or vice versa). Although the empirical relationship between theta power and memory is long established (Düzel et al., 2010), and experiments have shown memory impairments following the abolition of theta (McNaughton, Ruan, & Woodnorth, 2006; Winson, 1978), this is, to the best of our knowledge, the first model demonstrating a possible relation between theta frequency and memory, and thus providing a possible mechanistic role for the frequency perturbations caused by anxiolytics.

Importantly, our model additionally accounts for the empirical observation that theta oscillations coherent with hippocampal activity appear in the rodent LA after fear conditioning (Likhtik et al., 2014; Seidenbecher et al., 2003). The model posits that potentiated hippocampal inputs at theta frequency elicit firing in the LA, which collectively yields oscillations of the LFP at the same frequency. A similar phenomenon has been reported in the medial prefrontal cortex (mPFC) of rodents at the decision point of a maze after successful learning of task rules (Benchenane et al., 2010). This suggests that post‐learning theta synchronization might reflect functional pairing between the hippocampus and other brain regions.

We examined contextual fear conditioning because it can be more easily controlled in empirical studies and the underlying neural circuit is well‐described, whereas innate anxiety is likely to originate from hard‐wired adaptive tendencies with a partly unknown neural basis. However, we note that anxiolytics also affect behavior in a range of tests not involving conditioning (Choleris, Thomas, Kavaliers, & Prato, 2001; Pellow, Chopin, File, & Briley, 1985). For instance, the open field test is a common approach/avoidance anxiety test for rodents, consisting of an open arena in which the rodent can roam freely. The natural rodents' propensity toward exploring the arena conflicts with the preferential avoidance of exposed central regions, causing them to spend longer time along the walls in the periphery (Choleris et al., 2001). However, as in contextual conditioning, both the firing rate of LA neurons and avoidance behavior build up during exposure to the environment over minutes, suggesting that even so‐called innate anxiety behavior may arise from learning processes (Wang et al., 2011). Since the animal is usually placed in innate anxiety tests only once during their lifetime, it appears possible that they learn a cognitive map of the threatening environment features during the test. This learning might similarly be impaired by slowing theta frequency. Nonetheless, our model is not intended to address behavioral or neural responses associated with inherently aversive stimuli, which proceed in the absence of learning, although theta frequency coupling between medial temporal lobe regions during such experience may emerge as well (Zheng et al., 2017).

Our model predicts that the behavioral effect of anxiolytics originates from impairing the creation of neural associations between context and potential threat. This further predicts that anxiolytics do not impair already formed associations. In support of this, previous studies showed that benzodiazepines block fear conditioning if administered just before the conditioning epoch, but they are ineffective when administered just before recall (Sanger & Joly, 1985). This effect is not limited to learned anxiety. Multiple studies have shown that once animals experience the elevated plus‐maze without drug, anxiolytics are rendered ineffective in later exposure to the test—a phenomenon called “one trial tolerance” (File, 1993; File, Mabbutt, & Hitchcott, 1990).

Whether our model could also account for the clinical effect of anxiolytics—a reduction in subjective feelings of anxiety—remains unclear, as the neurobiological basis of these feelings is incompletely understood (Bach & Dayan, 2017; LeDoux, 2014). Importantly, we note that the anxiolytic effect in clinical contexts is immediate and does not vanish on repeated exposure to an anxiety‐generating context (Escarabajal, Torres, & Flaherty, 2003). It may therefore be that the drug effect on anxiety behavior, and subjective feelings of anxiety, are mediated by distinct mechanisms.

Importantly, however, the particular theta frequency values used in these simulations do not affect the qualitative nature of our results—that reductions in theta frequency will reduce synaptic potentiation and therefore impair learning. This is the case for any pair of (baseline and anxiolytic reduced) theta frequencies in the ~4–9 Hz range, below which no learning takes place, and above which increases in synaptic weight begin to saturate (as illustrated by Figure 2). Our model also suggests that larger theta reduction will cause a proportionally larger reduction in learning, although the downstream effect on the behavioral expression of freezing depends on the firing threshold that elicits freezing, which induces a non‐linearity.

A testable prediction of our model is that reduction of theta frequency alone, that is, without administration of anxiolytics, has an impact on learning and memory, and on anxiety behavior. To empirically demonstrate this, one could exploit the variability between epochs of theta activity and investigate correlations between theta frequency during conditioning and memory, as indexed by behavioral expression of anxiety during later recall. Other approaches require experimental manipulation of theta frequency. Previous studies have shown that this is possible by reducing the temperature of the brain (Whishaw & Vanderwolf, 1971). However, the fine frequency tuning necessary for probing the proposed effect might be difficult, and the secondary effects of temperature reduction cannot be ruled out easily. More recently, experimentally‐controlled theta oscillations have been induced with electrical patterned microstimulation (Lesting et al., 2011) and optogenetic approaches (Korotkova & Ponomarenko, 2017), providing viable methods for testing the proposed relation. This would also mitigate a concern that anxiolytic drugs have a wider array of effects beyond decreasing theta frequency, possibly encompassing an impact on nociception, which were not modeled here.

In summary, we presented a biophysical model suggesting that the behavioral effect of anxiolytics could be mediated by the effect that anxiolytics exert on theta frequency. The model accounts for a range of experimental findings and makes novel predictions about the effect of theta frequency on contextual fear conditioning, which may be tested in future experimental investigations.

## Data Availability

Data sharing is not applicable to this article as no new data were created or analysed in this study. The codes used in this study can be downloaded from github.com/gxcastegnetti/plathe.
